# Effects of exercise interventions on bone health and body composition in postmenopausal women with breast cancer: a systematic review and meta-analysis

**DOI:** 10.3389/fonc.2025.1593165

**Published:** 2025-06-18

**Authors:** Shan Yu, Chengfei Gao, Chuanjian Yi, Jingyou Hei

**Affiliations:** ^1^ Department of Endocrinology, The Second People’s Hospital of Liaocheng, Liaocheng, Shandong, China; ^2^ Department of Rehabilitation Medicine, The Affiliated Hospital of Qingdao University, Qingdao, Shandong, China; ^3^ Department of Joint Surgery, The Second People’s Hospital of, Liaocheng, Shandong, China

**Keywords:** exercise intervention, breast cancer, bone mineral density, body composition, meta - analysis

## Abstract

**Objective:**

To evaluate the effects of exercise interventions on bone health and body composition in postmenopausal women with breast cancer.

**Methods:**

A systematic search was conducted across PubMed, EMBASE, Web of Science, CENTRAL, and CNKI databases for randomized controlled trials (RCTs) published before October 2024. Data from eligible studies were extracted and analyzed using STATA software.

**Results:**

Eight RCTs comprising 1099 participants were included. The results indicated no significant differences between exercise and control interventions in patients’ bone mineral density (BMD) at the lumbar spine (WMD = 0.116, 95% CI [-0.357, 0.589], p = 0.631), femoral neck (WMD = -0.214, 95% CI [-0.497, 0.068], p = 0.137), or total hip (WMD = 0.299, 95% CI [-0.283, 0.882], p = 0.314). For body composition parameters, exercise interventions led to significant improvements in lean mass (WMD = 0.192, 95% CI [0.023, 0.362], p = 0.026) and marked reductions in percent body fat (WMD = -1.327, 95% CI [-2.587, -0.066], p = 0.039) compared to the control. However, no significant differences were observed in body weight (WMD = -0.024, 95% CI [-0.193, 0.146], p = 0.784) or fat mass (WMD = -0.078, 95% CI [-0.703, 0.546], p = 0.806) between the two interventions.

**Conclusion:**

The current evidence suggested that exercise interventions effectively improve lean mass and reduce percent body fat but have a limited impact on BMD in postmenopausal women with breast cancer. A multimodal, individualized exercise program is recommended to address the challenges of bone health and body composition in this population.

**Systematic review registration:**

https://www.crd.york.ac.uk/prospero/, identifier CRD42024613744.

## Introduction

Breast cancer is the most commonly diagnosed cancer among women globally, with postmenopausal women accounting for a substantial proportion of cases (1). Significant progress in treatment, including advancements in early detection and the development of more effective therapies, has led to a marked improvement in breast cancer survival rates (2). However, many breast cancer survivors continue to endure long-term side effects from treatments such as aromatase inhibitors and chemotherapy. These therapies are often linked to an increased risk of osteoporosis as well as adverse changes in body composition (3, 4). Such changes heighten the risks of fractures, metabolic disorders, and cardiovascular diseases, ultimately leading to a decline in overall life quality of affected individuals (5, 6).

In postmenopausal women, the natural decline in estrogen levels accelerates bone loss, a process further compounded by breast cancer treatments (7). Consequently, conditions such as osteopenia and osteoporosis are prevalent among these women, significantly increasing the risk of fractures and functional impairment (8). Besides concerns related to bone health, a concurrent loss of muscle mass and increase in fat mass are commonly observed, leading to diminished physical function and elevated risk of cardiovascular complications (9, 10). These adverse effects highlight the urgent need for effective interventions to mitigate the negative impact of breast cancer treatments on bone health and body composition, particularly in postmenopausal women.

Exercise is increasingly recognized as an effective non-pharmacological intervention to enhance bone health and counteract adverse changes in body composition among breast cancer survivors (11). Specifically, resistance and weight-bearing exercises have been shown to help maintain or improve bone mineral density (BMD) and preserve lean muscle mass, while aerobic exercises are associated with reductions in body fat (9, 12). Despite these promising effects, evidence regarding the efficacy of various types of exercise interventions for postmenopausal women with breast cancer remains inconsistent. While some studies report significant benefits, others indicate minimal or no effect (13, 14).

To address these inconsistencies, a systematic review and meta-analysis is necessary to synthesize the existing evidence on the effects of exercise on bone health and body composition in postmenopausal women with breast cancer. This review aims to provide robust, evidence-based support for the clinical application of exercise as an intervention for this population, thereby guiding healthcare providers and informing future treatment strategies.

## Method

The present study was conducted in accordance with the Preferred Reporting Items for Systematic Reviews and Meta-Analyses (PRISMA) guideline (15). The protocol was registered in PROSPERO with the registration number CRD42024613744.

### Eligibility criteria

The PICOS framework were applied to determine study eligibility (16). Studies were included if they involved postmenopausal women diagnosed with breast cancer (Population), who participated in exercise interventions such as aerobic, resistance, or weight-bearing training (Intervention), with a comparison group that received either no exercise or usual care (Comparison). The outcomes of interest were changes in bone mineral density and/or body composition (Outcome). Only randomized controlled trials (Study design) were considered for inclusion, with no restrictions on language. Studies were excluded if they involved premenopausal women, patients with major comorbid conditions affecting bone health or body composition, or if they evaluated non-exercise interventions. Additionally, retrospective trials, case reports, and conference papers were excluded.

### Search strategy

An electronic search of the PubMed, EMBASE, Web of science, CENTRAL and CNKI databases was performed to identify potentially relevant studies published before October 2024. Search strategies were customized for each database using a combination of medical subject headings (MeSH) and free-text keywords. For instance, in PubMed, the following search strategy was applied: ((“postmenopausal women”[MeSH] OR “postmenopausal”[tiab]) AND (“breast cancer survivors”[tiab] OR “breast neoplasms”[MeSH] OR “breast cancer”[tiab])) AND (“exercise”[MeSH] OR “physical activity”[tiab] OR “resistance training”[MeSH] OR “aerobic exercise”[MeSH] OR “weight-bearing exercise”[tiab]) AND (“bone mineral density”[MeSH] OR “BMD”[tiab] OR “bone health”[tiab] OR “body composition”[MeSH] OR “lean mass”[tiab] OR “fat mass”[tiab] OR “body weight”[tiab] OR “ percent body fat “[tiab]) AND (randomized controlled trial[pt] OR controlled clinical trial[pt] OR randomized[tiab] OR placebo[tiab] OR “randomly”[tiab] OR trial[tiab]) NOT (animals[mh] NOT humans[mh]). For the CNKI database, a combination of Chinese equivalents for the following terms was used: “postmenopausal,” “breast cancer,” “exercise,” “bone mineral density,” and “body composition.” Boolean operators such as “AND” and “OR” were applied to build the search strategy. Both subject terms and free-text keywords were utilized to ensure a comprehensive and sensitive search. Reference cited in all included studies were also manually examined for additional records.

### Study screening and data extraction

Two reviewers independently screened and assessed whether the retrieved studies met the inclusion criteria. The following information was then extracted from each eligible study: first author, publication year, participant characteristics, exercise intervention details, control condition, outcome measures, and follow up duration. Any discrepancies in data extraction were resolved through consensus with a third reviewer.

### Study quality assessment

The methodological quality of the included studies was assessed by two independent reviewers using the Cochrane Collaboration’s tool (17). The following aspects were evaluated as either low, unclear or high risk of bias: (1) random sequence generation, (2) allocation concealment, (3) blinding of participants and researchers, (4) blinding of outcome assessment, (5) incomplete outcome data, (6) selective reporting, and (7) other bias. Any disagreements between the reviewers were resolved through discussion consensus with a third reviewer.

### Statistical analysis

Weighted mean difference (WMD) and 95% confidence interval (CI) were pooled to calculate effect sizes, as the included studies reported statistics and variances in consistent units. Between-study heterogeneity was assessed using the I^2^ statistic, where values of 50% serve as a cutoff for low and high heterogeneity levels (18). A fixed-effect model was applied in the absence of significant heterogeneity, whereas a random-effect model was used otherwise. A funnel plot and Egger’s test were employed to detect potential publication bias, provided that a sufficient number of studies was available (19). All statistical analyses were conducted using Stata software 16.0 (StataCorp LP, College Station, TX), with a p-value <0.05 considered statistically significant.

## Results

### Study selection

**Figure 1:**
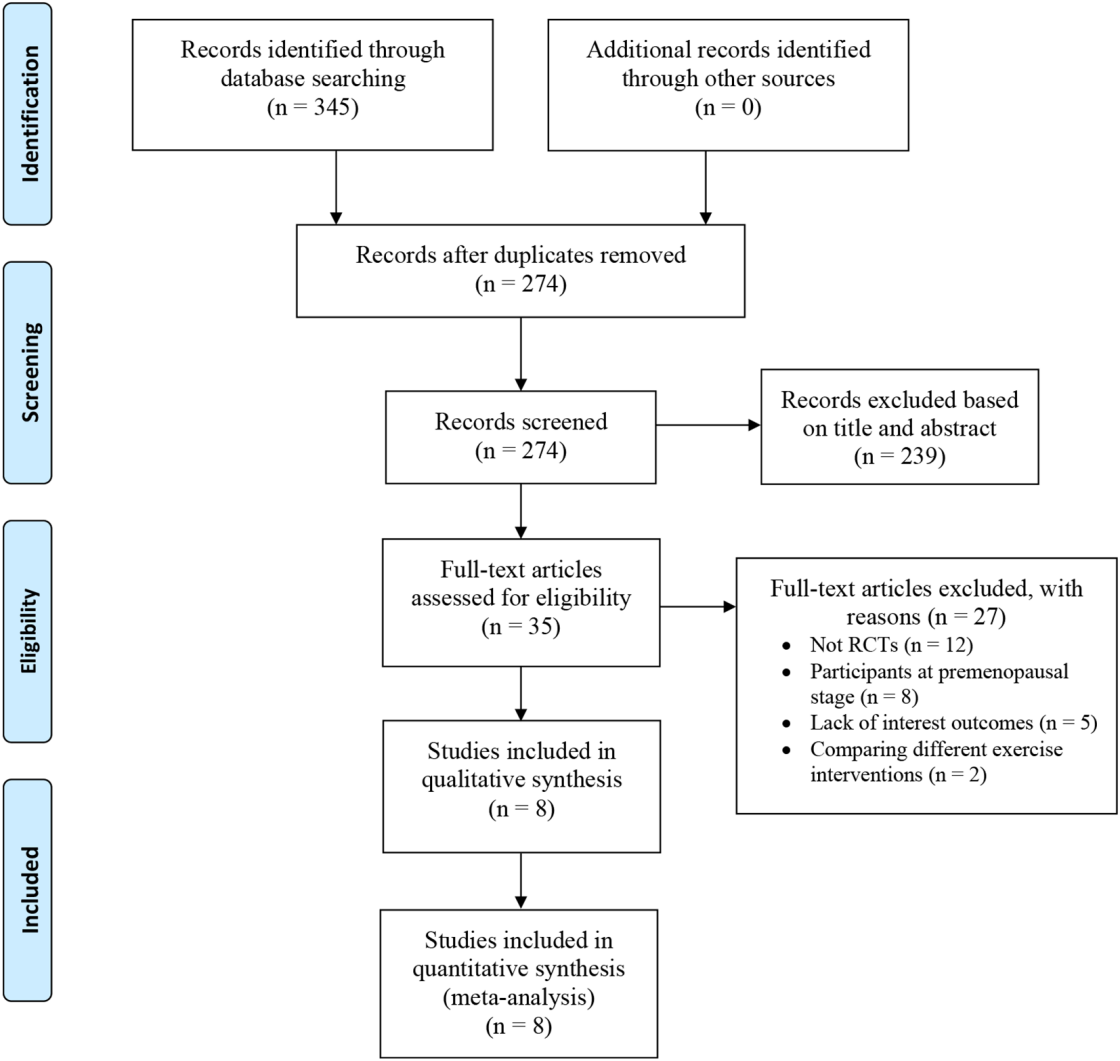
PRISMA flow diagram of study selection.

A total of 345 potentially relevant studies were identified in the preliminary search. After duplicated records were removed, 274 titles and abstracts were reviewed and screened for inclusion. Following the filtering process, 35 full-text articles were assessed for eligibility. Twenty-seven studies were excluded for the following reasons: 12 were non-randomized controlled studies, 8 included participants at the premenopausal stage, 5 lacked the outcomes of interest, and 2 compared different exercise interventions. Finally, 8 studies were included for data extraction and final meta-analysis (20–26). The study selection process, conducted based on the PRISMA statement was demonstrated in **Figure 1**.

### Study characteristics

The included studies were published between 2009 and 2023, involving a total of 1099 participants. All participants were breast cancer survivors at various stages (0–III), with mean ages ranging from 50.6 to 63.12 years. There was notable heterogeneity in the exercise intervention programs across the studies. While most control group participants received usual care or engaged in usual activities (20–26), only one study employed stretching training as the control intervention (9). Bone mineral density was reported in the majority of studies, with measurements taken at the lumbar spine (21–23, 25, 26), femoral neck (9, 21–23, 25, 26), and total hip (9, 21, 22, 25). Body composition outcomes, including body weight (9, 20, 23, 24), lean mass (9, 20, 23, 24), fat mass (9, 23, 24), and percent body fat (9, 20, 24) were evaluated in five out of eight studies. Follow-up durations varied, ranging from 6 to 24 months, with most studies lasting 12 months. **Table 1** illustrated the detailed characteristics of eligible studies.

**Table 1:** The characteristics of eligible studies.

Author/year	Participants no. (I/C)	Age, mean year, (I/C)	Cancer stage	Exercise intervention details					Control condition	Outcomes	Follow-up duration
				Type	Time/session	Frequency	Intensity	Duration			
Irwin et al. (20), 2009	37/38	56.5/55.1	stage I–III	aerobic training	15–30 minutes	three times weekly	60–80% PMHR	5 weeks	usual activities	body weight; lean mass; percent body fat	12 months
Kim et al. (21), 2016	23/20	55.7/56.3	stage I–III	aerobic and resistance training	15–30 minutes	twice to three times weekly	11-13 RPE	6 months	usual care	BMD (lumbar spine, femoral neck, total hip)	6 months
Knobf et al. (22), 2016	62/63	50.6/53.1	NA	aerobic and resistance training	10–45 minutes	three times weekly	50–70% PMHR	6 months	usual care	BMD (lumbar spine, femoral neck, total hip)	12 months
Saarto et al. (23), 2012	138/131	58/58	NA	aerobic and resistance training	60 minutes	once weekly	14–16 RPE	12 months	usual activities	BMD (lumbar spine, femoral neck); body weight; lean mass; percent body fat	12 months
Tang et al. (26), 2023	90/86	63.12/62.48	NA	aerobic training	50 minutes	five times weekly	70% PMHR	12 months	usual care	BMD (lumbar spine, femoral neck)	12 months
Thomas et al. (24), 2017	60/61	62.0/60.5	stage I–III	aerobic and resistance training	150 minutes	twice weekly	60–80% PMHR	12 months	usual activities	body weight; lean mass; fat mass; percent body fat	12 months
Waltman et al. (25), 2010	110/113	NA	stage 0-II	resistance training	30–45 minutes	twice weekly	NA	24 months	usual care	BMD (lumbar spine, femoral neck, total hip)	24 months
Winters-Stone et al. (9), 2011	36/31	62.3/62.2	stage 0-IIIA	resistance and weight bearing training	45–60 minutes	twice weekly	NA	12 months	stretching training	BMD (lumbar spine, femoral neck, total hip); body weight; lean mass; fat mass; percent body fat	12 months

BMD, bone mineral density; I/C, intervention/control; NA, not available; PMHR, predicted maximal heart rate; RPE, rating of perceived exertion.

### Study quality assessment

**Figure 2:**
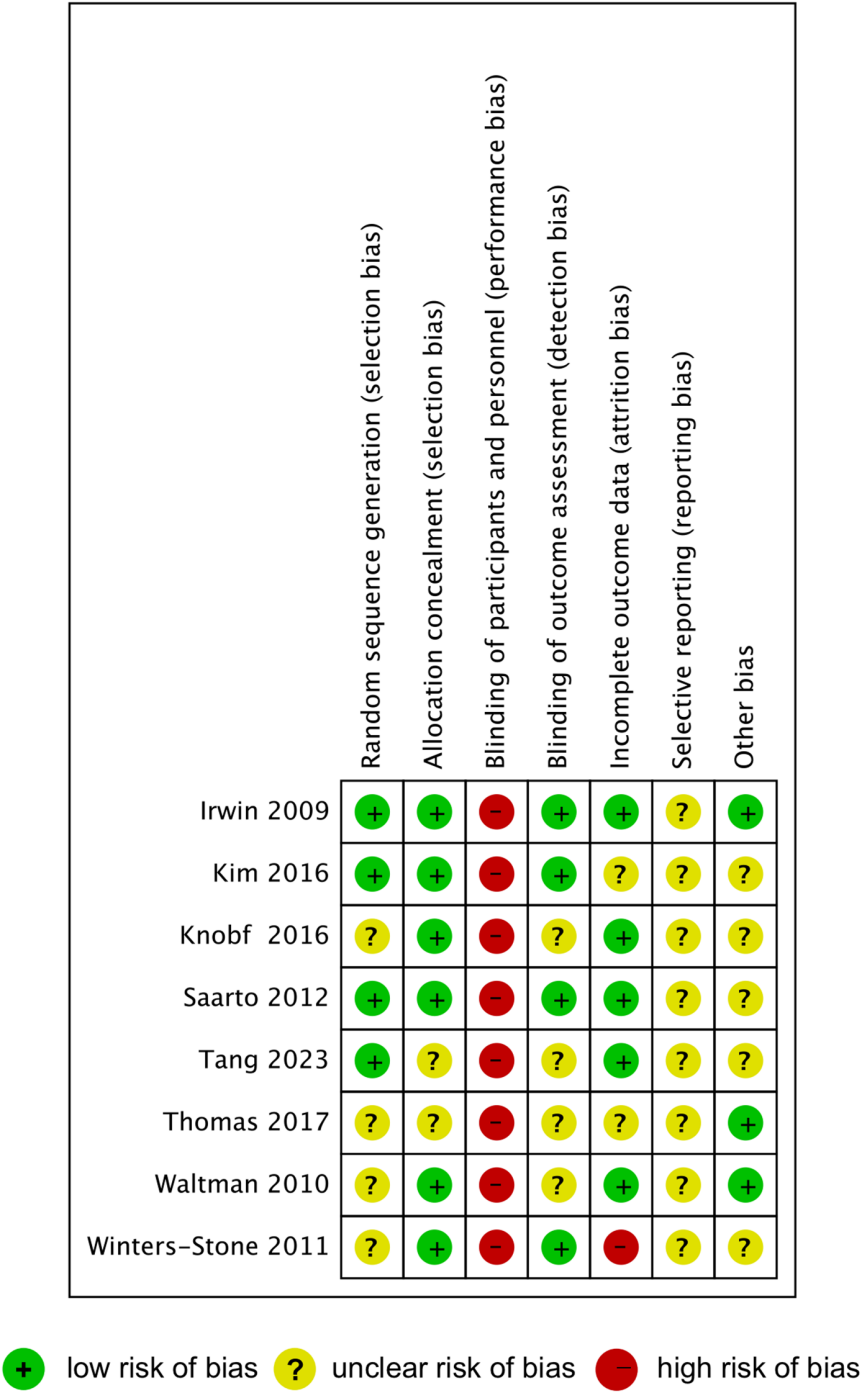
Quality assessment of the studies included in this meta-analysis.

The results of quality assessment of the included studies were summarized in **Figure 2**. Four studies were rated as low risk for random sequence generation, while the other four studies were rated as unclear risk. Allocation concealment bias was generally low, with only two studies showing unclear risks. High risk of bias was observed across all studies in the blinding of participants and personnel, as exercise interventions inherently prevent effective blinding. Half of the included studies were classified as low risk in the blinding of outcome assessment, while the other half were categorized as unclear risk. Incomplete outcome data were assessed as low risk in five studies, unclear risk in two studies, and high risk in one study. Most studies demonstrated an unclear risk for selective reporting and other biases, except for two studies rated as low risks.

### Meta-analysis

#### Effect of exercise on bone mineral density

**Figure 3:**
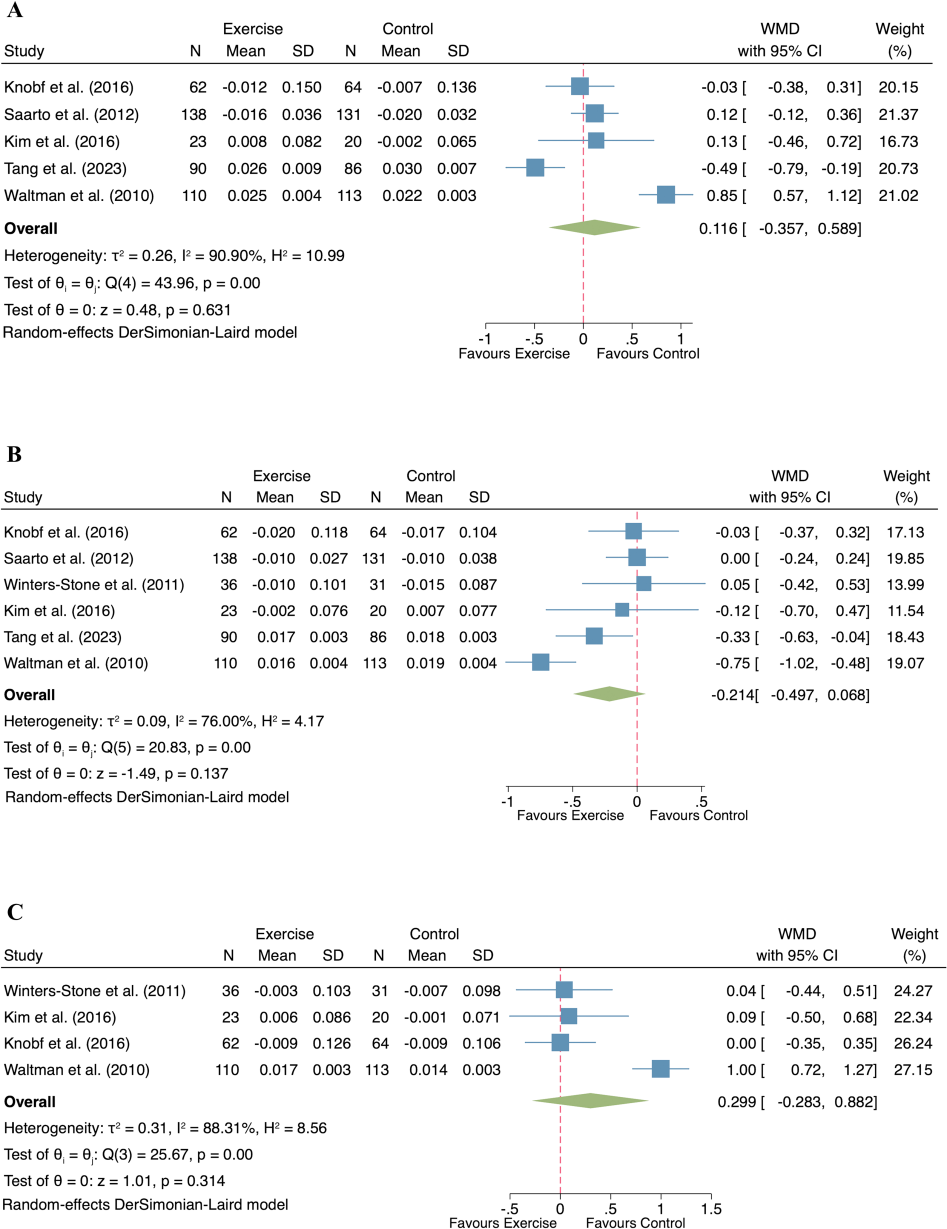
Forest plot of exercise compared to control on bone mineral density in postmenopausal women with breast cancer. (**(A)** Lumbar spine; **(B)** Femur neck; **(C)** Total hip).

Five studies (n= 423 participants) investigated the effect of exercise on lumbar spine BMD in postmenopausal women with breast cancer. A random-effect model was used due to the observed heterogeneity (p < 0.001, I^2^ = 90.90%), and the exercise intervention did not result in a significant improvement in lumbar spine BMD compared to the control group (WMD = 0.116; 95% CI: -0.357 to 0.589; p = 0.631; **Figure 3A**).

The effect of exercise on femur neck BMD was evaluated in six studies comprising 459 participants. Given the observed heterogeneity (I² = 76.00%, p < 0.001), a random-effect model was applied. The pooled analysis showed no significant difference in femur neck BMD between the exercise and control groups (WMD = -0.214, 95% CI: -0.497 to 0.068, p = 0.137; **Figure 3B**).

Four studies, involving 231 breast cancer survivors, examined the influence of exercise on total hip BMD. With significant heterogeneity detected (I² = 88.31%, p < 0.001), a random-effect model was applied. The meta-analysis found no statistically significant effect of exercise on total hip BMD in postmenopausal women (WMD = 0.299, 95% CI: -0.283 to 0.882, p = 0.314; **Figure 3C**).

#### Effect of exercise on body composition

**Figure 4:**
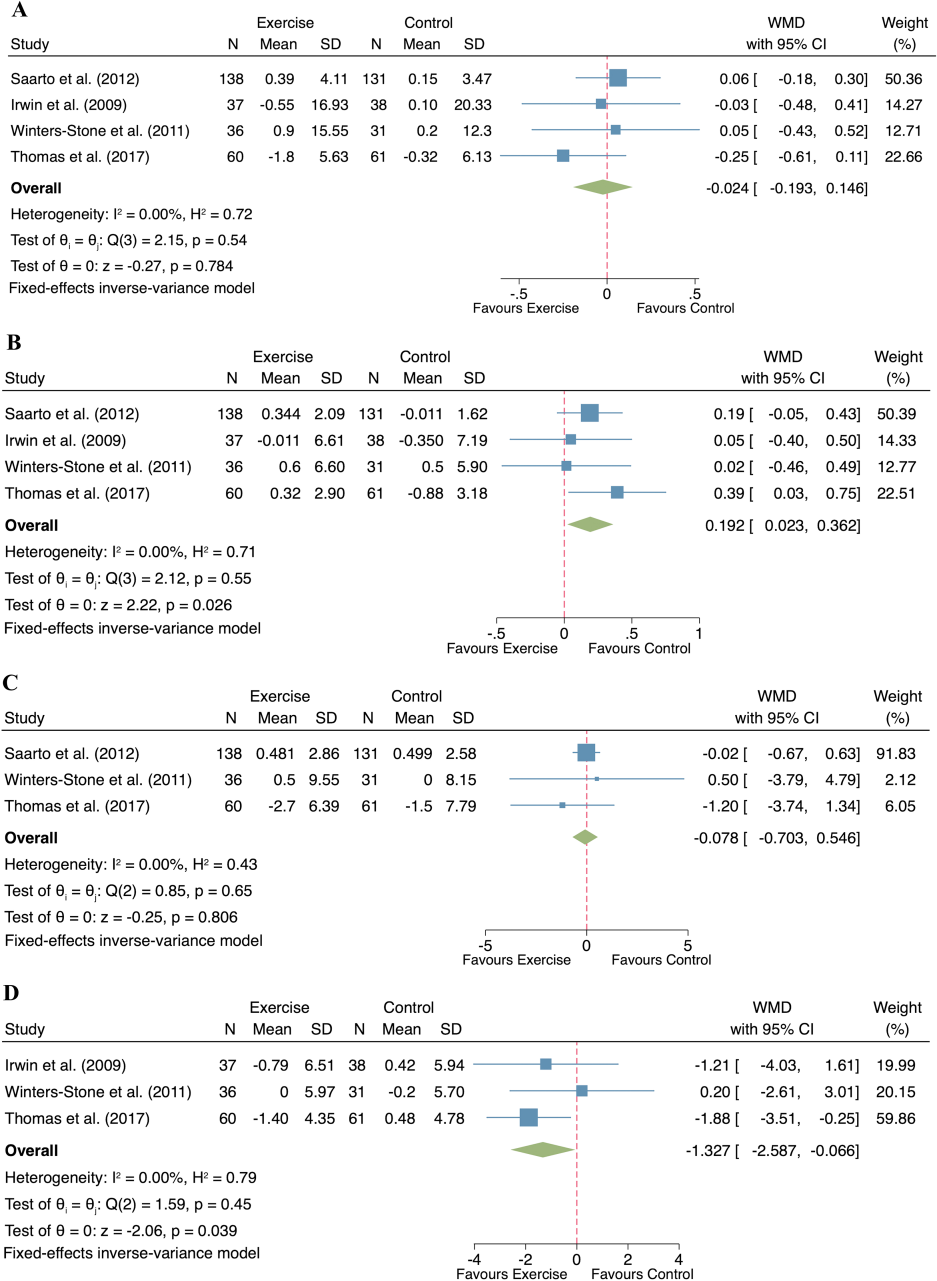
Forest plot of exercise compared to control on body composition in postmenopausal women with breast cancer. (**(A)**. Body weight; **(B)**. Lean mass; **(C)**. Fat mass; **(D)**. Percent body fat).

A total of four studies reported the effects of exercise on body composition in postmenopausal women with breast cancer (n= 271 participants) across four indicators: body weight, lean mass, fat mass and percent body fat. No significant heterogeneity was observed for any of the outcomes (body weight: I² = 0%, p = 0.54; lean mass: I² = 0%, p = 0.55; fat mass: I² = 0%, p = 0.65; percent body fat: I² = 0%, p = 0.45), justifying the application of fixed-effect models. The exercise intervention did not result in significant changes in body weight (WMD = -0.024, 95% CI: -0.193 to 0.146, p = 0.784; **Figure 4A**) and fat mass (WMD = -0.078, 95% CI: -0.703 to 0.546, p = 0.806; **Figure 4C**) compared to the control group. However, significant improvements in lean mass (WMD = 0.192, 95% CI: 0.023 to 0.362, p = 0.026; **Figure 4B**) and reductions in percent body fat (WMD = -1.327, 95% CI: -2.587 to -0.066, p = 0.039; **Figure 4D**) were evident in the exercise group compared to the control group.

### Publication bias

**Figure 5:**
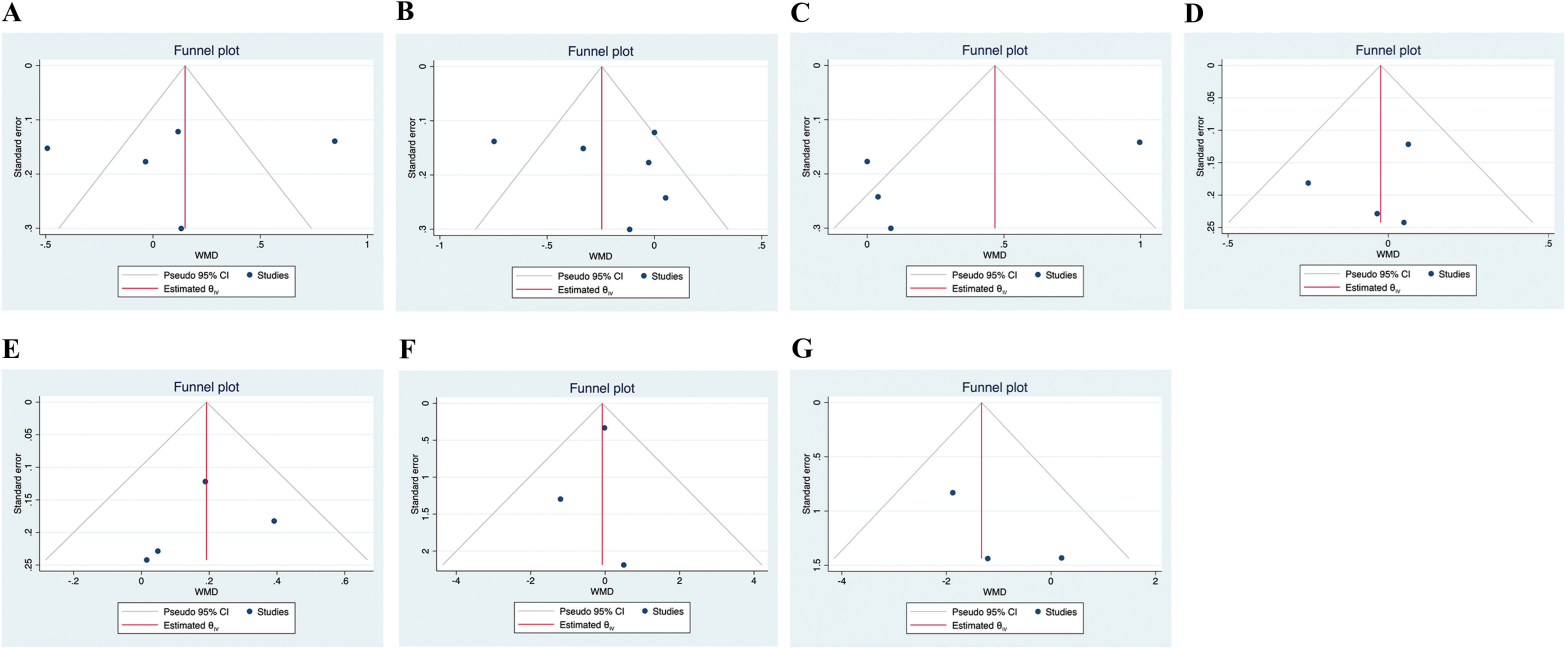
Funnel plots of the publication bias. (**(A)**. Lumbar spine BMD; **(B)**. Femur neck BMD; **(C)**. Total hip BMD; **(D)**. Body weight; **(E)**. Lean mass; **(F)**. Fat mass; **(G)**. Percent body fat).

As shown in **Figure 5**, no significant publication bias was found according to the visualization of the funnel plot and the results from the Egger’s test. (p=0.881 for lumbar spine BMD; p=0.496 for femur neck BMD; p=0.279 for total hip BMD; p=0.613 for body weight; p=0.699 for fat mass; p=0.601 for lean mass; p=0.296 for percent body fat)

## Discussion

The primary finding of this meta-analysis was that exercise interventions did not significantly improve BMD at key anatomical sites, including the lumbar spine, femoral neck, and total hip, in postmenopausal women with breast cancer. This lack of improvement in BMD with exercise aligns with previous research, such as the meta-analysis by Fornusek et al., which highlighted the limited osteogenic effect of exercise among postmenopausal breast cancer survivors (27). The postmenopausal status of participants, combined with the use of aromatase inhibitors (AIs), likely contributes to this limited response (28). AIs exacerbate estrogen deficiency, common in this population, which leads to increased bone resorption and diminishes the effectiveness of exercise as an osteogenic stimulus (29, 30). Although resistance or weight-bearing exercises generally provide mechanical loading that promotes bone formation (31), the hormonal imbalance resulting from reduced estrogen levels predisposes individuals to bone degradation, thereby limiting the efficacy of exercise in increasing BMD (32). Nevertheless, it is essential to note the substantial heterogeneity in our meta-analysis results, which may limit the strength of these conclusions. Possible explanations for this heterogeneity include variations in exercise types and intensities, participant characteristics such as baseline BMD and AI usage, diverse intervention durations affecting detectable bone changes, and methodological differences in BMD measurement. These considerations highlight the complexity involved in evaluating exercise effects on bone health and underscore the necessity for standardized exercise protocols and longer intervention periods in future studies. In contrast, evidence from real-world data by Abdel-Razeq et al. demonstrated that a comprehensive approach combining exercise with pharmacological interventions, such as bisphosphonates, is more effective in maintaining or improving BMD (13). Bisphosphonates suppress osteoclast activity, reduce bone resorption, and thus complement the osteogenic effects of exercise (33). This synergistic approach emphasizes the need for a multimodal strategy, where exercise serves as an adjunct to pharmacotherapy rather than as a standalone treatment to counteract bone loss in this population.

Beyond the effects on bone mineral density, this meta-analysis also assessed the impact of exercise interventions on body composition in postmenopausal women. The results indicated a reduction in percent body fat and an increase in lean mass, while total fat mass and body weight remained largely unchanged. These findings are consistent with previous studies, which reported that exercise interventions, particularly resistance exercise, enhances muscle mass and reduces fat percentage in breast cancer survivors, even though overall body weight often remains stable (34, 35). Although exercise significantly improved lean mass and reduced percent body fat, the absence of significant changes in total body weight and fat mass may reflect a recomposition effect, where muscle gain offsets fat loss, resulting in minimal net weight change (36). Furthermore, most included studies did not incorporate dietary control, which likely limited the potential for fat mass reduction, as exercise alone may not induce sufficient energy deficit (37). In contrast, the improvements in lean mass can be attributed to the anabolic effects of resistance or combined training, promoting muscle hypertrophy via increased protein synthesis (36). The reduction in percent body fat may also reflect enhanced metabolic efficiency, including improved insulin sensitivity and greater fat oxidation (12). This consistency underscores that exercise interventions primarily enhance physical fitness and body composition quality, as evidenced by increased lean mass and reduced body fat percentage, which are crucial for improving quality of life and reducing the risk of disease recurrence in breast cancer survivors (38, 39).

This study has several limitations. First, although substantial heterogeneity was observed in several pooled outcomes, subgroup or sensitivity analyses were not feasible due to the limited number of included studies and the inconsistent reporting of participant and intervention characteristics. This limitation should be considered when interpreting the meta-analytic results. Second, the relatively small sample sizes in several trials may have reduced the statistical power of the pooled estimates. Third, the duration of most interventions was relatively short (≤12 months), which may be insufficient to produce detectable changes in bone mineral density. Fourth, all included studies were rated as high risk for performance bias due to the inability to blind participants and personnel in exercise trials. This may have introduced subjective bias, particularly for self-reported outcomes such as body composition. However, BMD was objectively measured using standardized methods, which partially mitigates this concern. Finally, this review was based solely on quantitative data from randomized controlled trials. Future research incorporating qualitative approaches is needed to better understand patients’ individual needs and preferences, which may inform the design of more personalized and effective exercise interventions.

## Conclusion

In conclusion, exercise interventions provide notable benefits for body composition but have limited impact on BMD in postmenopausal women with breast cancer. A comprehensive approach that integrates pharmacological treatments with individualized exercise programs may provide an optimal strategy for enhancing overall health outcomes in this population. These findings emphasize the necessity of multimodal and personalized interventions that address the diverse health challenges faced by breast cancer survivors, with a particular focus on preserving bone health while improving body composition.

## Data Availability

The raw data supporting the conclusions of this article will be made available by the authors, without undue reservation.

## References

[1] Bray F Ferlay J Soerjomataram I Siegel RL Torre LA Jemal A . Global cancer statistics 2018: GLOBOCAN estimates of incidence and mortality worldwide for 36 cancers in 185 countries. CA Cancer J Clin. (2018) 68:394–424. doi: 10.3322/caac.21492 30207593

[2] Chen X Hu G Yu Q . Impact of disulfidptosis-associated clusters on breast cancer survival rates and guiding personalized treatment. Front Endocrinol (Lausanne). (2023) 14:1256132. doi: 10.3389/fendo.2023.1256132 38116315 PMC10728640

[3] Pedersini R Schivardi G Laini L Zamparini M Bonalumi A di Mauro P . Changes in body composition in early breast cancer patients treated with aromatase inhibitors. J Endocrinol Invest. (2024) 47:3119–28. doi: 10.1016/j.breast.2024.103794 PMC11549134 38856966

[4] Nisha Y Dubashi B Bobby Z Sahoo JP Kayal S Ananthakrishnan R . Negative impact on bone homeostasis in postmenopausal women with non-metastatic breast cancer during cytotoxic chemotherapy. J Bone Miner Metab. (2023) 41:682–92. doi: 10.1007/s00774-023-01444-9 37410202

[5] Pandey P Sharma A Gogia A . Bone health in breast cancer. Curr Probl Cancer. (2023) 47:100959. doi: 10.1016/j.currproblcancer.2023.100959 37236055

[6] Patnaik JL Byers T DiGuiseppi C Dabelea D Denberg TD . Cardiovascular disease competes with breast cancer as the leading cause of death for older females diagnosed with breast cancer: a retrospective cohort study. Breast Cancer Res. (2011) 13:R64. doi: 10.1186/bcr2901 21689398 PMC3218953

[7] Cucciniello L Garufi G Di Rienzo R Martinelli C Pavone G Giuliano M . Estrogen deprivation effects of endocrine therapy in breast cancer patients: Incidence, management and outcome. Cancer Treat Rev. (2023) 120:102624. doi: 10.1016/j.ctrv.2023.102624 37751658

[8] Rizzoli R Bischoff-Ferrari H Dawson-Hughes B Weaver C . Nutrition and bone health in women after the menopause. Womens Health (Lond). (2014) 10:599–608. doi: 10.2217/WHE.14.40 25482487

[9] Winters-Stone KM Dobek J Nail L Bennett JA Leo MC Naik A . Strength training stops bone loss and builds muscle in postmenopausal breast cancer survivors: a randomized, controlled trial. Breast Cancer Res Treat. (2011) 127:447–56. doi: 10.1007/s10549-011-1444-z PMC3124708 21424279

[10] Silverman SL Delmas PD Kulkarni PM Stock JL Wong M Plouffe L Jr . Comparison of fracture, cardiovascular event, and breast cancer rates at 3 years in postmenopausal women with osteoporosis. J Am Geriatr Soc. (2004) 52:1543–8. doi: 10.1111/j.1532-5415.2004.52420.x 15341559

[11] Ficarra S Thomas E Bianco A Gentile A Thaller P Grassadonio F . Impact of exercise interventions on physical fitness in breast cancer patients and survivors: a systematic review. Breast Cancer. (2022) 29:402–18. doi: 10.1007/s12282-022-01347-z PMC9021138 35278203

[12] Courneya KS Segal RJ Mackey JR Gelmon K Reid RD Friedenreich CM . Effects of aerobic and resistance exercise in breast cancer patients receiving adjuvant chemotherapy: a multicenter randomized controlled trial. J Clin Oncol. (2007) 25:4396–404. doi: 10.1200/JCO.2006.08.2024 17785708

[13] Abdel-Razeq H Al-Rasheed U Mashhadani N Al-Ibraheem A Abdel-Razeq R Jaradeh SA . The efficacy of a comprehensive bone health program in maintaining bone mineral density in postmenopausal women with early-stage breast cancer treated with endocrine therapy: real-world data. Ir J Med Sci. (2022) 191:2511–5. doi: 10.1007/s11845-021-02897-5 35088228

[14] Gonzalo-Encabo P Valadés D García-Honduvilla N de Cos Blanco A Friedenreich CM Pérez-López A . Exercise type and fat mass loss regulate breast cancer-related sex hormones in obese and overweight postmenopausal women. Eur J Appl Physiol. (2020) 120:1277–87. doi: 10.1007/s00421-020-04361-1 32266494

[15] Page MJ McKenzie JE Bossuyt PM Boutron I Hoffmann TC Mulrow CD . The PRISMA 2020 statement: an updated guideline for reporting systematic reviews. Bmj. (2021) 372:n71. doi: 10.1136/bmj.n71 33782057 PMC8005924

[16] Amir-Behghadami M Janati A . Population, Intervention, Comparison, Outcomes and Study (PICOS) design as a framework to formulate eligibility criteria in systematic reviews. Emerg Med J. (2020) 37:387. doi: 10.1136/emermed-2020-209567 32253195

[17] Higgins JP Altman DG Gøtzsche PC Jüni P Moher D Oxman AD . The Cochrane Collaboration’s tool for assessing risk of bias in randomised trials. Bmj. (2011) 343:d5928. doi: 10.1136/bmj.d5928 22008217 PMC3196245

[18] Higgins JP Thompson SG Deeks JJ Altman DG . Measuring inconsistency in meta-analyses. Bmj. (2003) 327:557–60. doi: 10.1136/bmj.327.7414.557 PMC192859 12958120

[19] Pustejovsky JE Rodgers MA . Testing for funnel plot asymmetry of standardized mean differences. Res Synth Methods. (2019) 10:57–71. doi: 10.1002/jrsm.v10.1 30506832

[20] Irwin ML Alvarez-Reeves M Cadmus L Mierzejewski E Mayne ST Yu H . Exercise improves body fat, lean mass, and bone mass in breast cancer survivors. Obes (Silver Spring). (2009) 17:1534–41. doi: 10.1038/oby.2009.18 PMC2841468 19629060

[21] Kim SH Cho YU Kim SJ Hong S Han MS Choi E . The effect on bone outcomes of adding exercise to supplements for osteopenic breast cancer survivors: A pilot randomized controlled trial. Cancer Nurs. (2016) 39:144–52. doi: 10.1097/NCC.0000000000000245 25730596

[22] Knobf MT Jeon S Smith B Harris L Kerstetter J Thompson AS . Effect of a randomized controlled exercise trial on bone outcomes: influence of adjuvant endocrine therapy. Breast Cancer Res Treat. (2016) 155:491–500. doi: 10.1007/s10549-016-3693-3 26850265

[23] Saarto T Sievänen H Kellokumpu-Lehtinen P Nikander R Vehmanen L Huovinen R . Effect of supervised and home exercise training on bone mineral density among breast cancer patients. A 12-month randomised controlled trial. Osteoporos Int. (2012) 23:1601–12. doi: 10.1007/s00198-011-1761-4 21892676

[24] Thomas GA Cartmel B Harrigan M Fiellin M Capozza S Zhou Y . The effect of exercise on body composition and bone mineral density in breast cancer survivors taking aromatase inhibitors. Obes (Silver Spring). (2017) 25:346–51. doi: 10.1002/oby.21729 PMC5450163 28026901

[25] Waltman NL Twiss JJ Ott CD Gross GJ Lindsey AM Moore TE . The effect of weight training on bone mineral density and bone turnover in postmenopausal breast cancer survivors with bone loss: a 24-month randomized controlled trial. Osteoporos Int. (2010) 21:1361–9. doi: 10.1007/s00198-009-1083-y 19802506

[26] Tang LC Sun J Jing YY Li L . Factors influencing the reduction of bone mineral density in postmenopausal breast cancer patients treated with endocrine therapy and analysis of the efficacy of aerobic exercise intervention. Chongqing Med J. (2023) 52:1384–9. doi: 10.3969/j.issn.1671-8348.2023.09.021

[27] Fornusek CP Kilbreath SL . Exercise for improving bone health in women treated for stages I-III breast cancer: a systematic review and meta-analyses. J Cancer Surviv. (2017) 11:525–41. doi: 10.1007/s11764-017-0622-3 28639157

[28] Kharb R Haider K Neha K Yar MS . Aromatase inhibitors: Role in postmenopausal breast cancer. Arch Pharm (Weinheim). (2020) 353:e2000081. doi: 10.1002/ardp.202000081 32449548

[29] Kim C Na Y Lee S Park JY Chung YJ Song J . A recent review of the management of postmenopausal symptoms in breast cancer survivors. J Menopausal Med. (2023) 29:85–91. doi: 10.6118/jmm.23016 38230591 PMC10796204

[30] Gallagher JC . Role of estrogens in the management of postmenopausal bone loss. Rheum Dis Clin North Am. (2001) 27:143–62. doi: 10.1016/S0889-857X(05)70191-5 11285992

[31] Dieli-Conwright CM Courneya KS Demark-Wahnefried W Sami N Lee K Sweeney FC . Aerobic and resistance exercise improves physical fitness, bone health, and quality of life in overweight and obese breast cancer survivors: a randomized controlled trial. Breast Cancer Res. (2018) 20:124. doi: 10.1186/s13058-018-1051-6 30340503 PMC6194749

[32] Ramaswamy B Shapiro CL . Osteopenia and osteoporosis in women with breast cancer. Semin Oncol. (2003) 30:763–75. doi: 10.1053/j.seminoncol.2003.08.028 14663777

[33] Bonaiuti D Shea B Iovine R Negrini S Robinson V Kemper HC . Exercise for preventing and treating osteoporosis in postmenopausal women. Cochrane Database Syst Rev. (2002) 3:Cd000333. doi: 10.1002/14651858.CD000333 12137611

[34] Al-Mhanna SB Batrakoulis A Norhayati MN Mohamed M Drenowatz C Irekeola AA . Combined aerobic and resistance training improves body composition, alters cardiometabolic risk, and ameliorates cancer-related indicators in breast cancer patients and survivors with overweight/obesity: A systematic review and meta-analysis of randomized controlled trials. J Sports Sci Med. (2024) 23:366–95. doi: 10.52082/jssm PMC11149074 38841642

[35] Fraser SF Gardner JR Dalla Via J Daly RM . The effect of exercise training on lean body mass in breast cancer patients: A systematic review and meta-analysis. Med Sci Sports Exerc. (2022) 54:211–9. doi: 10.1249/MSS.0000000000002792 34559724

[36] Yang H Liu L Zhang X . Exercise interventions on body composition and quality of life of overweight/obese breast cancer survivors: a meta-analysis. BMC Womens Health. (2023) 23:484. doi: 10.1186/s12905-023-02627-2 37700300 PMC10498647

[37] Brown JC Sarwer DB Troxel AB Sturgeon K DeMichele AM Denlinger CS . A randomized trial of exercise and diet on body composition in survivors of breast cancer with overweight or obesity. Breast Cancer Res Treat. (2021) 189:145–54. doi: 10.1007/s10549-021-06284-7 PMC8316406 34089422

[38] Shaikh H Bradhurst P Ma LX Tan SYC Egger SJ Vardy JL . Body weight management in overweight and obese breast cancer survivors. Cochrane Database Syst Rev. (2020) 12:Cd012110. doi: 10.1002/14651858.CD012110 33305350 PMC8094215

[39] Zagalaz-Anula N Mora-Rubio MJ Obrero-Gaitán E Del-Pino-Casado R . Recreational physical activity reduces breast cancer recurrence in female survivors of breast cancer: A meta-analysis. Eur J Oncol Nurs. (2022) 59:102162. doi: 10.1016/j.ejon.2022.102162 35716452

